# Contributing Factors and Induced Outcomes of Psychological Stress Response in Stroke Survivors: A Systematic Review

**DOI:** 10.3389/fneur.2022.843055

**Published:** 2022-06-22

**Authors:** Song Zhang, Yuan Yuan, Wenwen Zhuang, Tianqing Xiong, Yijun Xu, Jingwen Zhang, Chunhua Tao, Jingyan Liang, Yingge Wang

**Affiliations:** ^1^Department of Neurology, Affiliated Hospital of Yangzhou University, Yangzhou, China; ^2^School of Nursing and School of Public Health, Yangzhou University, Yangzhou, China; ^3^Institute of Translational Medicine, Medical College, Yangzhou University, Yangzhou, China; ^4^Jiangsu Key Laboratory of Integrated Traditional Chinese and Western Medicine for Prevention and Treatment of Senile Diseases, Yangzhou University, Yangzhou, China

**Keywords:** stroke, psychological stress, risk factor, outcome, perceived stress, post-traumatic stress disorder

## Abstract

**Background:**

Remarkable evidence indicates that psychological stress is significantly associated with stroke. However, a uniform recommendation to identify and alleviate poststroke psychological stress responses and improve postmorbid outcomes is not currently available. Thus, this systematic review aimed to summarize the types of poststroke psychological stress, measurement tools, contributing factors, and outcomes.

**Methods:**

This systematic review was undertaken in accordance with the Preferred Reporting Items for Systematic Reviews and Meta-Analyses. A literature search was conducted in PubMed, Web of Science, Embase, CNKI, WanFangData, and CQVIP from database inception to November 2021. Cross-sectional and longitudinal studies were included in this research. Quality assessment was performed based on the National Institutes of Health (NIH) Quality Assessment Tool for Observational Cohort and Cross-Sectional Studies.

**Results:**

Eighteen quantitative, peer-reviewed studies were included for analysis. Selected articles mainly investigated perceived stress and posttraumatic stress disorder after stroke. We classified the contributing factors into four categories: sociodemographic factors, clinical disease factors, psychological factors, and behavioral and lifestyle factors. The postmorbid outcomes were divided into three categories: clinical disease outcomes, psychological outcomes, and behavioral and quality of life outcomes.

**Conclusions:**

Compared to common patients, stroke survivors with the following characteristics suffered an increased psychological stress response: younger age, the presence of caregivers, depression, unsuitable coping strategies, etc. Meanwhile, lower quality of life, worse drug compliance, worse functional independence, and more severe mental disorders were significantly associated with increased psychological stress symptoms. Further studies are required to provide more trustworthy and meaningful references for mitigating the damage caused by psychological stress after stroke.

## Introduction

Stroke is the third leading cause of death and disability worldwide (1). In recent decades, the numbers of new first-time stroke victims and stroke survivors have separately increased by 68% and 84%, respectively, worldwide (2). Although the mortality from stroke has declined, stroke survivors still suffer from severe physical and psychological sequelae, which become a great burden to society and their families. Numerous studies have identified that stroke survivors experience short- and long-term depression, anxiety, and psychological stress (3). However, most studies were devoted to poststroke depression and anxiety, and studies investigating psychological stress after stroke are still needed.

Psychological stress response refers to the changes in negative emotional and functional states when individuals lack the resources and abilities to deal with threats (4). A major event such as a stroke is a stressor that makes survivors feel stressed. Related studies have shown that more than 33% of stroke survivors suffered from clinically significant psychological stressors (5, 6), and over 23% of them developed posttraumatic stress symptoms 1 year after onset (7). New studies have appeared in recent years as people's focus has gradually turned to poststroke psychological stress. Currently, the poststroke psychological stress response is mainly divided into two categories for research, namely, perceived psychological stress and posttraumatic stress symptoms (PTSS), also known as posttraumatic stress disorder (PTSD). The two categories have no compliance and will appear following the environmental and individual differences of patients at different stages of stroke.

There is no doubt that stroke can be detrimental to a person's life, while the psychological stress response will amplify and aggravate such negative effects (4, 8, 9). Furthermore, the psychological stress response, impacted by social factors and psychological comorbidities, has a similar or even more serious influence on mental health and the quality of life among survivors compared with physical disorders (10, 11). Thus, it is crucial to explore which factors can predict the psychological stress response of patients and its impact on the prognosis of stroke.

In recent years, there have been some cross-sectional and longitudinal studies on poststroke psychological stress symptoms, including analyses of early predictive factors and late prognostic effects. Nevertheless, most current reviews have focused on the prevalence of poststroke psychological stress response or mainly analyzed the predictors or prognostic effects of one type of response (5, 12, 13). Hence, this study attempts to integrate the existing relevant literature to elaborate on the status quo and assessment tools between two types of psychological stress responses after stroke and weigh the conflicting findings in premorbid predicting factors and postmorbid outcomes of psychological stress response to provide a reference for establishing a more effective intervention management strategy.

## Methods

### Eligibility Criteria

This systematic review was undertaken in accordance with the Preferred Reporting Items for Systematic Reviews and Meta-Analyses (14) (Supplementary Table 1). The authors declare that all supporting data are publicly available and appropriately cited within the article. After the removal of duplicate studies, two reviewers independently screened the titles, abstracts, and full-text copies to determine the final study inclusion. Disagreements were resolved through discussion, and a third reviewer assisted in providing confirmation when necessary. Eligible studies were assessed based on the predetermined inclusion and exclusion criteria.

The inclusion criteria were as follows: (1) studies with samples of adults aged older than 18 years with a history of stroke; (2) studies with patients who had experienced poststroke psychological stress, and the authors reported at least one related outcome; (3) studies in which the authors declared at least one measurement tool, predictive factor, or prognostic outcome of psychological stress; and (4) the cross-sectional studies, the longitudinal studies, the cohort studies, and case–control studies were included.

The exclusion criteria were as follows: (1) studies with mixed measurements of psychological stress (unless psychological stress measurements could be extracted); (2) studies with sample sizes <20; and (3) reviews, systematic reviews, meta-analyses, case reports, letters, protocols, conference abstracts, and unpublished studies.

### Search Strategy

For this systematic review, researchers performed a comprehensive search of English databases, including PubMed, EMBASE, Web of Science, CNKI, WanFangData, and CQVIP, from database inception to November 2021. All databases were searched without further limitations. Medical subject headings were applied based on MeSH and Entry Terms in PubMed, which were also modified and converted for other databases, maximizing citation retrieval.

To identify the related articles as comprehensively as possible, we conducted a systematic search of six databases. The following search terms were utilized: (Stroke OR Acute Cerebrovascular Accident OR Brain Vascular Accidents OR Apoplexy, Cerebrovascular, etc.) AND (Stress, Psychological OR Life Stress OR Stressor, Psychological, etc.), and the detailed search strategy is indicated in Supplementary Table 2.

### Data Extraction and Quality Appraisal

One reviewer extracted the data using Microsoft Excel 2019, and a second reviewer independently checked the extracted data. The extracted data included details of the study design, study purposes, setting, sample characteristics (number, age, sex, time since diagnosis, disease type), types of psychological stress, psychological stress measurement/scales, characteristics related to psychological stress reported in the results sections and main conclusions related to psychological stress reported in the articles. Methodological quality was assessed using the National Institutes of Health Quality Assessment Tool for Observational Cohort and Cross-Sectional Studies. The articles included in our research were rated as “good” or “fair.” Each study was evaluated by two independent reviewers, and a third reviewer was consulted when necessary.

### Synthesis of Results

The synthesis of the studies was presented in the form of a narrative review. In cases where the evidence was scarce, the evidence was briefly summarized to most accurately represent the current evidence.

## Results

### Study Selection

**Figure 1:**
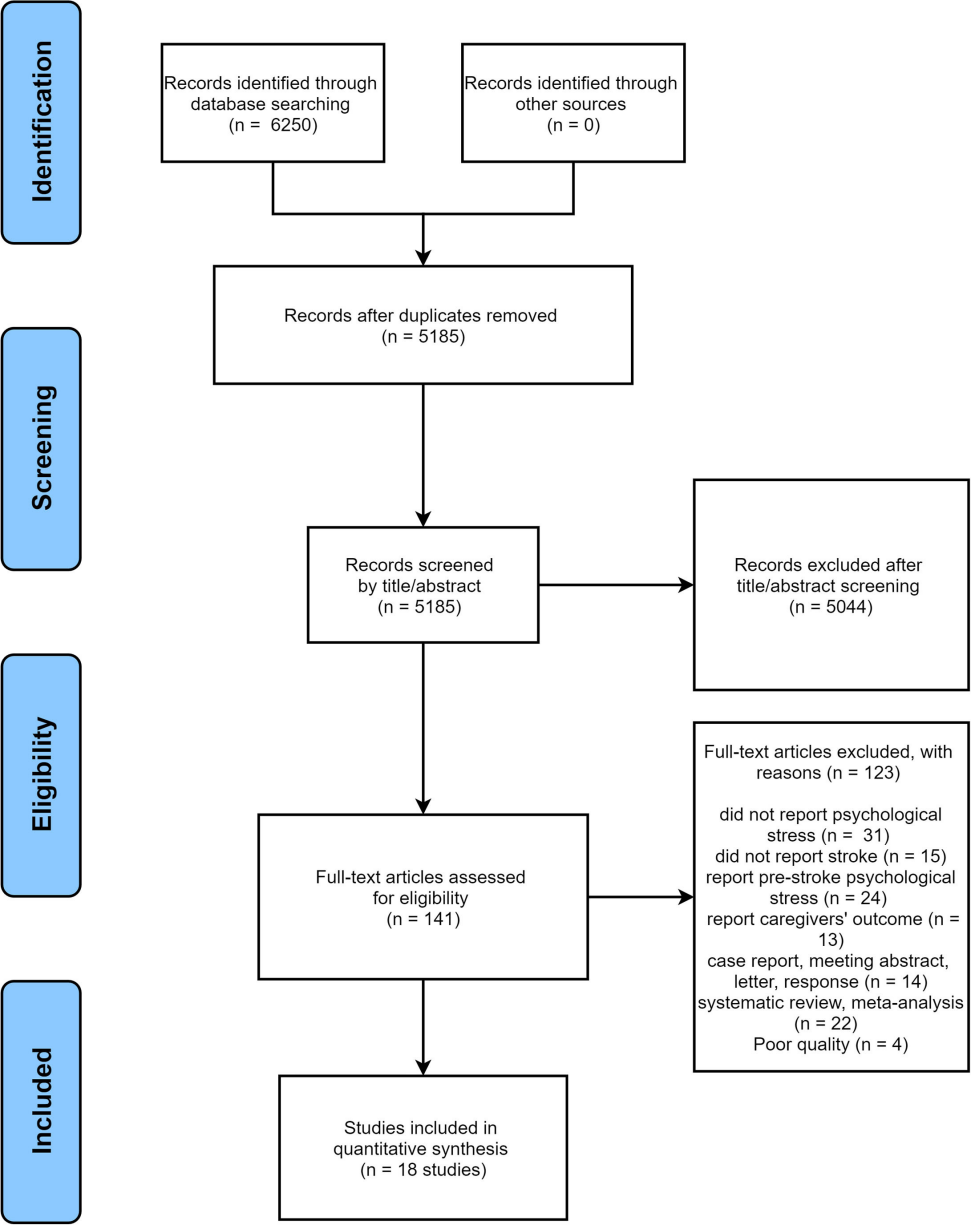
Flow diagram of study selection.

According to the research process, a total of 6,250 records were retrieved, of which 1,065 were deleted because of duplication. Then, the remaining 5,185 articles were screened via titles and abstracts, resulting in the exclusion of 5,044 studies. Finally, we conducted a full-text review of 141 papers. Of these, 123 were evaluated as unsuitable due to various reasons, and 18 met the inclusion criteria and were subsequently included. The flow diagram of study selection is presented in Figure 1.

### Study Characteristics

The 18 studies included 5,038 participants, of which 48.27% were male. The study designs were either cross-sectional studies (*n* = 12) or longitudinal studies (*n* = 6) containing cohort studies (*n* = 4). The locations of the studies were mainly in Brazil (*n* = 2), the United States (*n* = 7), Italy (*n* = 1), Jordan (*n* = 1), China (*n* = 5), Australia (*n* = 1), and Germany (*n* = 1). In addition, in terms of time since diagnosis, stroke survivors between 1 and 12 months after onset (*n* = 12) received more attention, while there were few studies on patients within 1 month (*n* = 5) or more than 12 months (*n* = 1) after stroke (Tables 1, 2). More data extraction information is presented in Supplementary Tables 3, 4.

**Table 1:** Different types and measurement methods of psychological stress after stroke and its premorbid predicting factors.

**Studies**	**Type of study**	**Main psychological stress measurements**	**Predicting factors**
dos Santos EB (15)	A longitudinal survey, quantitative	The Perceived Stress Scale−10 items (PSS-10)	Presence of caregivers, depression and lower functional independence
Cornelius (7)	A cohort survey, quantitative	Acute Stress Disorder Scale−19 items (ASDS) Post-traumatic stress disorder (PTSD) checklist for DSM-5 (PCL-5)	Presence of caregivers
Gandolfi (16)	A cross-sectional survey, quantitative	The Symptom Checklist-90 scale (SCL-90-R)	Suffer chronic pain
Garton (17)	A cohort survey, quantitative	Post-traumatic stress disorder (PTSD) checklist−17 items (PCL-17)	Younger age and worse functional outcomes
dos Santos et al. (2)	A cross-sectional survey, quantitative	The Perceived Stress Scale−10 items (PSS-10)	Depression and lower functional independence
Almhdawi et al. (11)	A cross-sectional survey, quantitative	Depression Anxiety Stress Scale−21 items (DASS-21)	Worse functional outcomes, discontinuation of rehabilitation services, female, and self-reported physical diseases other than stroke
Jiang et al. (18)	A cohort survey, quantitative	Impact of Event Scale-Revised−22 items (IES-R)	Female, worse functional outcomes, depression, and anxiety
Ostwald et al. (19)	A cross-sectional survey, quantitative	The Perceived Stress Scale−10 items (PSS-10)	Poorer function, depression and emotional distress
Huafang (20)	A longitudinal survey, quantitative	The Perceived Stress Scale−10 items (PSS-10)	Depression and worse functional outcomes
Ruan Wei (21)	A cross-sectional survey, quantitative	Post-traumatic stress disorder (PTSD) checklist−17 items (PCL-17)	Worse mental health and functional outcomes

**Table 2:** Different types and measurement methods of psychological stress after stroke and its effects on the postmorbid outcomes.

**Studies**	**Type of study**	**Main psychological stress measurements**	**Post-morbid outcomes**
Juth et al. (22)	A cross-sectional survey, quantitative	Acute stress disorder scale−14 items (ASDS) Post-traumatic stress disorder (PTSD) checklist for DSM-5 (PCL-5)	Worse psychological/emotional outcomes
Stein et al. (23)	A cross-sectional survey, quantitative	Post-traumatic stress disorder (PTSD) checklist-−17 items (PCL-17)	Depression, anxiety, worse quality of life, and worse functional outcomes
Müller et al. (24)	A cross-sectional survey, quantitative	Primary Care-PTSD Screen−4 items (PC-PTSD)	Depression and anxiety
Gyawali et al. (4)	A cross-sectional survey, quantitative	The Perceived Stress Scale−10 items (PSS-10)	Lower levels of resilience, higher levels of functional impairments, and worse psychological/emotional outcomes
Guo et al. (8)	A cross-sectional survey, quantitative	The Perceived Stress Scale−10 items (PSS-10)	Depression
Laures-Gore et al. (9)	A cohort survey, quantitative	The Perceived Stress Scale−10 items (PSS-10)	Depression
Kronish et al. (25)	A cross-sectional survey, quantitative	Post-traumatic stress disorder (PTSD) checklist−17 items (PCL-17)	Non-adherent to medications, depression, and worse cognitive function
Aixia et al. (26)	A cross-sectional survey, quantitative	The Perceived Stress Scale−14 items (PSS-14)	Depression

### Risk of Bias

According to the National Institutes of Health (NIH) Quality Assessment Tool for Observational Cohort and Cross-Sectional Studies and the discussion of the three reviewers, 9 studies each were classified as having “Good” (8, 11, 15, 17, 19, 20, 24–26) and “Fair” (2, 4, 7, 9, 16, 18, 21–23) methodological qualities (Supplementary Tables 5, 6). The evaluation of the risk of bias revealed that the sample size justification was the weakest aspect, and only 6.67% of the studies reported how the sample size was determined; thus, it was not clear whether the sample size was sufficient in the studies. Moreover, regarding the repeated measures, blinding methods, and pre-exposure measurements, since both cross-sectional and longitudinal studies were included, the reported results were classified as “fair” based on the research structure. Finally, the measurement tools, sample attrition, and analysis domains were reported well.

### Types of Psychological Stress

After the collation and analysis of literature data, 10 and 8 articles were separately analyzed to research the predictive factors and prognosis of stroke. Among these, studies based on perceived psychological stress tended to analyze the predictors of psychological stress, while those based on PTSD tended to analyze the prognosis of patients. In the case of study design, only one longitudinal study analyzed the perceived psychological stress after stroke, and the other 7 studies that analyzed this were cross-sectional studies. Nevertheless, poststroke PTSD was researched using 4 cross-sectional studies and 3 longitudinal studies, including 2 cohort studies.

### Measurement Tools

#### Perceived Stress Scale

The perceived stress scale (PSS) (2, 4, 8, 9, 15, 19, 20, 26) is an instrument that is widely applied for evaluating the degree of perceived psychological stress in stroke survivors. More specifically, this scale, in accordance with the stress-coping framework of McCubin (19), mainly focuses on the unpredictability, uncontrollability, and overload of psychological stress (8, 15). The PSS is divided into two dimensions: the sense of not being in control and the feeling of tension. In addition, this instrument is scored using a 5-point Likert scale with higher scores indicating more severe psychological stress. Its item scores are defined as 1 (strongly disagree) to 5 (strongly agree). The PSS is available in 10- and 14-item versions, which both have high internal reliability. At present, the PSS has been utilized in stroke patients and identified to have acceptable psychometric properties (15, 27). The Cronbach's α of the PSS ranges from 0.83 to 0.88.

The other remaining tools used to assess perceived psychological stress include the Symptom Checklist-90 (SCL-90-R) scale (11) and the Depression Anxiety Stress Scale (DASS-21) (19). The SCL-90-R contains 90 items that are rated on a 5-point Likert scale ranging from “not at all” to “extremely.” The DASS-21 was designed to measure depression, anxiety, and stress via 7 items for each symptom (28). Among these, scores of 9, 7, and 14 were independently allocated to depression, anxiety, and stress, respectively. Higher DASS-21 scores indicate more serious symptoms, and perceived psychological stress is one of the measured variables.

#### Posttraumatic Stress Disorder Checklist

The posttraumatic stress disorder checklist (PCL) (7, 17, 21–23, 25) is a scale including two forms, the 17-item version and the 20-item version, that has been extensively validated and utilized as an indicator of PTSD (5, 29). All the items are related to a potentially traumatic event and are consistent with the Diagnostic and Statistical Manual of Mental Disorders 4th Edition (DSM-IV) criteria. This instrument is also scored using a 5-point Likert scale, and the item responses range from 1 (not at all) to 5 (extremely). The total scores, ranging from 17 to 85, indicate PTSD when the score is over 44 points (30). The scale should usually be applied, at least, a minimum of 1 month after onset. Furthermore, this scale has two forms with 17 items and 20 items. The Cronbach's α of the PCL ranges from 0.92 to 0.96 (7, 22).

All the other scales that are applied for poststroke PTSD include the Acute Stress Disorder Scale−14 items (ASDS) (7, 22), Impact of Event Scale-Revised−22 items (IES-R) (18), and Primary Care-PTSD Screen-4 items (PC-PTSD) (24). The ASDS is often used to evaluate early symptoms relevant to the development of PTSD and possesses strong internal consistency and test–retest reliability (31, 32). This scale includes 14 items and total scores range from 14 to 70. The response options range from 1 (not at all) to 5 (very much). Higher scores indicate more serious symptoms. The Cronbach's α of the ASDS ranges from 0.85 to 0.93 (7, 22). The IES-R is utilized to measure the following representative symptom clusters of PTSD: intrusion (8 items), avoidance (8 items), and hyperarousal (6 items) (18). Every item is rated from 0 (not at all) to 4 (extremely), and the identification of PTSD requires a total score of over 30 points (33). The PC–PTSD is an extensively validated 4-item scale and is consistent with the DSM-IV. In addition, its optimally sensitive score was 3 (34).

### Premorbid Predicting Factors

After summarizing the 10 included articles related to the premorbid predictive factors of psychological stress response, we mainly classified the factors into four categories: sociodemographic factors, clinical disease factors, psychological factors, and behavioral and lifestyle factors.

### Sociodemographic Factors

Age, sex, income, education, and comorbidities were identified to be significantly correlated with poststroke perceived psychological stress after univariate analysis. A relevant study (15) indicated that women were more common than men in terms of the distribution of perceived psychological stress. Another study (2) reported the opposite result: sex was not a contributing factor to psychological stress. In addition, three studies (2, 15, 19) drew the reverse conclusions with respect to age and comorbidity. Of these, age and comorbidities were identified to be irrelated to psychological stress response (2), while the other two studies showed that young patients and patients with more comorbidities were considered to be at risk for a more severe psychological stress response (15, 19). According to the multiple regression analyses, age was also included in the model as a significant predictor (19).

Age, sex, education level, marital status, income level, work status, comorbidities, length of hospitalization, and religious status were analyzed to explore their relevance to stroke survivors suffering from poststroke PTSD. Garton et al. considered that young patients were associated with a higher psychological stress response (14), while Jiang C thought that women and religious status were connected with a higher psychological stress response (18). However, education level, marital status, income level, work status, comorbidities, and length of hospitalization were identified to have no relevance to stress (17, 18). Notably, regarding age and sex, these two articles obtained opposite results (17, 18).

### Clinical Disease Factors

Poststroke psychological stress has been reported to be associated with clinical disease factors. At present, neurological deficits, chronic pain, functional independence, and grip strength have been shown to be relevant to perceived psychological stress (2, 11, 15, 16, 19, 21). Worse neurological deficits, more severe chronic pain, lower functional independence, and weaker grip strength suggested higher psychological stress symptoms. Gandolfi et al. (16) found that psychological stress and chronic pain influenced each other. The former predicted the latter, while the latter, in turn, affected the former. Multiple regression models showed that functional independence and grip strength were contributing factors to psychological stress (2, 11, 15, 19).

With respect to poststroke PTSD, functional independence, the location and size of the hematoma, premorbid function, the prognosis of early stroke, the degree of handicap, and cognitive function have been evaluated (17, 18). The results indicated that only lower functional independence and a higher degree of handicap could predict PTSD after stroke onset. However, premorbid function, the prognosis of early stroke, cognitive function, and the location and size of the hematoma were not contributing factors to PTSD after stroke.

### Psychological Factors

Many psychological factors are common causes of the poststroke psychological stress response. For instance, depression, anxiety, emotion, mental health, and self-reported health status were significantly connected with perceived psychological stress. Of these, higher scores of mental health symptoms, such as depression and anxiety, and worse self-reported health status corresponded with higher perceived stress, which were also effectually included in the multiple regression model (2, 11, 15, 19, 20).

In addition, threat awareness, depression, and anxiety were also explored to verify their impact on PTSD after stroke. Studies have shown that stronger threat awareness and more serious depression and anxiety are obvious risks of psychological stress (7, 18).

### Behavioral and Lifestyle Factors

There is no doubt that behavior and lifestyle can influence stroke survivors' psychological stress symptoms. Studies have indicated that the presence of caregivers, daily daytime sleeping hours, and rehabilitation are contributing factors to perceived psychological stress (2, 11, 15). These accompanied with the presence of caregivers, shorter sleep hours, and the continuation of rehabilitation enhanced perceived stress. These three factors were also validated in the multiple regression model (2, 11). However, the opposite conclusion existed that rehabilitation had no effect on stress (15).

In terms of PTSD after stroke, the presence of caregivers and unsuitable coping strategies were identified as contributing factors. It has been verified that the presence of caregivers and unsuitable coping strategies effectively increase poststroke psychological stress symptoms (7, 18).

### Postmorbid Outcomes

After summarizing the 8 included articles related to the postmorbid outcomes of psychological stress response, we mainly classified them into the following three categories: clinical disease outcomes, psychological outcomes, and behavioral and quality of life outcomes.

### Clinical Disease Outcomes

A related study suggested that the relationship between perceived psychological stress and poststroke functional outcomes did not appear to vary with the length of time poststroke (4). However, perceived psychological stress has proven to be negatively associated with poststroke outcomes based on the Stroke Impact Scale (SIS), including memory and thinking, mood and emotion, and participation/role function. In the adjusted multiple regression analysis, the correlation between stress and functional outcomes was unequivocal. Interestingly, the region of stroke was also analyzed in another article, and the results showed that the survivors who experienced a stroke in the left hemisphere perceived less stress than those who experienced a stroke in the right hemisphere (9).

With respect to poststroke PTSD, two articles drew two conclusions. In the early days after onset, PTSD was entirely unrelated to poststroke functional outcomes (24). In contrast, within 6–12 months after stroke, PTSD could predict the deterioration of functional independence after stroke (23).

### Psychological Outcomes

Perceived psychological stress was reported to be associated with poststroke depression, including direct and indirect effects (8, 9, 20). When perceived stress directly influenced depression, the multiple regression model showed that 67.4% of the variation in depression was explained by the independent variables. With a sense of coherence dimensions added to the model, the multiple linear regression model accounted for 71.6% of the variance in depression based on indirect effects. In addition, resilience has been identified to be negatively related to the psychological stress response, and both were independently associated with stroke outcomes (4).

Posttraumatic stress disorder had a substantial influence on psychological outcomes after stroke. On the one hand, PTSD, depression, and anxiety appeared to have a high degree of overlap after stroke (23). On the other hand, PTSD is significantly correlated with poststroke depression and anxiety in patients with PTSD alone (24, 25).

### Behavior and Quality of Life

At present, few studies have paid attention to the behavior or quality of life of stroke survivors with perceived psychological stress. No relevant reports were extracted from the articles we included. In terms of poststroke PTSD, relevant articles have shown that stroke survivors with PTSD suffer worse quality of life, and their rate of return to work dramatically declines (23). Moreover, the reduction in medication compliance also became the main outcome accompanied by poststroke PTSD. The results showed the grading correlation between PTSD and drug compliance, and the worse drug compliance was the more severe PTSD after onset (25). Notably, in the multiple regression models, quality of life and medication adherence were obviously influenced by PTSD (23, 25).

## Discussion

The poststroke psychological stress response has been determined to be detrimental to stroke survivors (4, 23, 25), and it is common, progressive, and far-reaching. Relevant articles have shown that it could cause changes in social function (35), mental health (36), and emotional behavior (37). Therefore, the aim of this review was to summarize the types, measurement tools, the premorbid predicting factors, and the postmorbid outcomes of poststroke psychological stress response, based on existing articles, to provide credible evidence for enabling the effective management of alleviating the symptoms of psychological stress and improving the prognosis of stroke.

In the 18 studies included in this systematic review, perceived psychological stress and PTSD were mainly discussed as the two types of poststroke psychological stress. Perceived psychological stress is defined as a particular relationship between an individual and their environment, where the individual considers himself or herself as requiring more resources to deal with existing or imminent threats (15). PTSD refers to a psychiatric disorder caused by a traumatic event with the characteristics of re-experiencing the event, negative changes in emotion, the avoidance of trauma, and hypervigilance (38). The existing articles found that the two types of poststroke psychological stress had overlapping parts to a certain extent (7, 22). Acute stroke patients suffer from high perceived stress and early posttraumatic stress symptoms. Stroke survivors with PTSD also experienced considerable stress in the chronic phase of stroke (4). However, the present study mainly focused on one type of psychological stress, and few studies have combined and generalized the two types of psychological stress after stroke to analyze the hierarchical correlation between them.

### Measurement Tools

The poststroke psychological stress response is a complicated subjective and multidimensional feeling. It is challenging to evaluate stress and transform it to quantitative data based on limited information. Thus, a suitable, available measurement tool is crucial to assess participants. In this review, we included two tools to assess perceived psychological stress and four tools to evaluate PTSD. We found that the PSS and PCL were the most utilized scales and were also widely used for cancer, migraine, cardiovascular diseases, etc. (39–41). However, these tools, used in different studies, are available in multiple versions with a diverse number of options, and no research can confirm that one version has been validated to be better than other tools for evaluating the psychological stress response in stroke patients. Thus, the reliability and validity of the PSS and its suitability for stroke survivors require further exploration. In addition, in terms of the PCL, the ASDS has been proven to be better than the PCL due to its design and structure. A relevant article reported that stroke patients were likely to experience “mental fog,” which made interviews beyond their ability, and a dichotomous survey shorter than 20 min led to better results (22). Hence, an analysis of the characteristics of stroke patients should also be included to determine better measurement tools and should not be limited to reliability and validity.

The primary outcome we explored in this review was to summarize the premorbid predicting factors and postmorbid outcomes of psychological stress after stroke.

### Premorbid Predicting Factors

In the first part, we analyzed the 8 included articles and divided the contributing factors into four major categories.

### Sociodemographic Factors

Poststroke psychological stress has been reported to be associated with sociodemographic factors. Younger age is a significant factor, which is attributed to the different concepts between young and elderly patients (42). Studies have shown that different age groups have various levels of perceived stress and that the elderly population thought that stroke was due to the increase in age (15, 43). Thus, higher psychological stress symptoms occur in young patients due to the lack of mental preparation. Based on the previous studies, we have found that the effect of gender on psychological stress remained controversial (12, 17, 23, 33). There have been studies where sex and psychological stress were the independent variables (44). Therefore, perhaps whether women are more susceptible to suffering psychological stress responses after stroke is worth further exploration in the future. The relationship between education level and psychological stress response has been demonstrated, and a low education level was a significant predictor of stress (17, 23). One reason could be better comprehension ability and high compliance with doctors. Nevertheless, the effect of education on stroke survivors was not clear in our included articles. Furthermore, Santos et al. (15) demonstrated that discharge policies in different countries and regions also had a significant impact on psychological stress. For example, patients in the United States were likely to be referred to inpatient rehabilitation facilities after the acute phase of stroke, while stroke survivors in Brazil tended to be transitioned to their homes regardless of their functional status, which might cause a large psychological gap for patients. Currently, there is a lack of research on this aspect. The remaining variables, such as income level, work status, comorbidities, and the length of hospitalization, which predict poststroke psychological stress are yet to be thoroughly determined (12, 17).

### Clinical Disease Factors

In this review, we found that regardless of the existence of perceived stress or PTSD after stroke, functional independence was always a significant predictor. Due to the different testing nodes, participants were in different phases of functional status, which influenced the results assessed. Stroke survivors in the acute stage tended to have worse functional outcomes than convalescent patients and suffered higher psychological stress (2, 17). Interestingly, researchers found that convalescent patients also showed different degrees of stress (45). Among these patients, survivors in the early stage paid more attention to athletic rehabilitation, while those in the chronic phase gradually turned their attention from rehabilitation to worries about relapse, death, and disability, which may partly explain this phenomenon. Moreover, we found that most studies did not analyze the influence of stroke types on psychological stress after stroke onset. However, there have been studies including different types of strokes or studies devoted to a certain type of stroke. Thus, it is necessary to study the effect of stroke classification on psychological stress (12).

### Psychological Factors

As indicated previously, psychological factors had a far-reaching influence on psychological stress after stroke. Currently, stroke rehabilitation is mainly focused on physical function, while less attention is given to psychological status (4). There have been many studies where functional outcomes influenced the psychological stress response (2, 15). However, Müller et al. considered that the degree of disability after an acute stroke had no consistency with psychological symptoms, which indicated that further observation of the psychological response in stroke survivors was necessary (24). Meanwhile, a related study showed that the relationship between functional outcomes and psychological stress after stroke could be mediated by the constellation of social factors, such as recovery status, social support, insurance status, etc. (17). Therefore, the specific mechanisms of the effect of functional status on the poststroke psychological stress deserve further exploration. In our included articles, many studies focused on the effect of stress on depression or anxiety, but the studies sometimes ignored the predictive effect of depression or anxiety on stress. Poststroke psychological symptoms are highly overlapped (23). Therefore, except for analyzing the impact of only one factor at a time, the influence of the interaction of psychosocial factors on PTSD is lacking and worth exploring (12). Previous studies outlined that a sense of coherence was a protective factor for PTSD in other diseases (46, 47). The sense of coherence as the mediator of the positive effect of mindfulness on post-TIA PTSD has been successfully applied. Currently, there are few studies on positive mental mediators of psychological stress responses after stroke, and this is worthy of exploration.

### Behavioral and Lifestyle Factors

Establishing a healthy lifestyle and a scientific recovery pattern are crucial for self-management in stroke survivors. Goldstein et al. and Sarchiapone et al. reported that there was a bidirectional and positive relationship between sleep and mood regulation (48, 49). Relevant studies have demonstrated that the decline in sleep hours obviously influences poststroke psychological stress symptoms (11). However, the specific correlation and mechanism of the impact of sleep on mental health after stroke have not been well analyzed. There is no doubt that social support plays an important role in the rehabilitation of stroke survivors, and the presence of caregivers is one of the crucial parts (38). Interestingly, contrary to conventional wisdom, caregivers do not always have a positive effect on psychological stress after stroke. Many studies have shown that caregivers have a negative influence on survivors (15, 45). The following three reasons might explain this phenomenon. First, caregivers may aggravate the perceived threats of patients in hospitals and exacerbate posttraumatic stress symptoms, which will increase psychological stress over time (7). Second, the excessive support of caregivers will bring patients into a state of life that does not match their functional independence and will also intensify their psychological stress. Third, the patients usually accepted psychological regulation, but the caregivers did not, so the caregivers faced more psychological pressure (50). However, studies have shown that the psychological stress of caregivers can be transmitted to patients, and the effect of intimate relationships is stronger than that of unfamiliar relationships (7, 45). Caregivers may also induce psychological stress in survivors, so it is important to establish a suitable care relationship.

### Postmorbid Outcomes

In the second part of this study, we analyzed the other 7 articles and divided the outcomes into three major categories.

### Clinical Disease Outcomes

As previously mentioned, the stress perception of patients who experienced a stroke in the right hemisphere was higher than that of patients who experienced a stroke in the left hemisphere over time (9). There were existing articles reporting the reverse conclusions (23, 51). However, functional independence has been demonstrated to be a significant predictor of psychological stress responses (4, 23, 24). Therefore, we suppose that patients with stroke pay more attention to changes in their ability to perform daily activities than to the abstract location of onset. Moreover, the measurement results were mainly presented as yes or no, and there is a lack of reports on severity classification (11). There is still a need for further research on the classification of various symptoms and their interactions.

### Psychological Outcomes

Psychological stress responses, depression, and anxiety have been proven to be highly overlapped in stroke survivors. At present, the existing studies extensively capture negative psychological states and explore their relevance (17). However, no study has explored comorbidities and their overlap (12), and distinguishing their differences and severities may be a reference research direction. Reports have shown that poststroke psychological stress is a predictive factor for poststroke depression (11, 38). Furthermore, stress can not only directly affect depression but can also indirectly influence depression via certain psychological responses (8). Therefore, combined with the above, the mechanism of the interaction between psychological stress and depression after stroke is worthy of further study.

### Behavior and Quality of Life

The negative mental status induced by stroke impedes the compliance of patients, which can even have an adverse impact on the caregivers' health (52). Interestingly, an article showed that the discontinuation of rehabilitation was positively related to the psychological stress response (11). Similarly, medication compliance was also influenced by poststroke psychological stress. Some articles indicated that drug efficacy was not effective in survivors with psychological stress, and the risk of stroke recurrence increased (6, 38). Thus, the cause of this phenomenon is probably that rehabilitation activities and drugs become reminders of trauma, which make patients stressed and avoidant. It may be worthwhile to analyze the extent to which rehabilitation activities and drugs remind patients of psychological stress for more convincing conclusions (53). In addition, psychological stress has been proven to be negatively related to the quality of life (23). In the existing studies, there is a lack of research on the quality of life, and the evaluation standard is not uniform (12).

### Limitations

This systematic review has several limitations. First, this article excluded gray literature due to the pragmatic limitations of resources, such as non-English publications and unpublished literature, which may extend related information about psychological stress symptoms. Second, only cross-sectional and longitudinal studies were included in this manuscript, and there were few randomized controlled trials for the psychological stress response after stroke. Therefore, more research should be conducted to further investigate the underlying mechanisms of the management of psychological stress. Third, this article generalized the different measurements for stress, and we did not consider the differences between various scales when analyzing the results related to psychological stress.

## Conclusion

The prevalence of poststroke psychological stress in stroke survivors may be associated with various aspects of predictive factors. Meanwhile, the outcomes induced by poststroke psychological stress in stroke survivors could also be divided into several categories. Thus, suitable measures can be applied to alleviate the psychological stress response after stroke based on these contributing factors and improve the prognosis of stroke according to the relationship between outcomes and poststroke stress. For instance, the relationship with the presence of caregivers, the high degree of overlap in mental illness, and the functional independence after onset should be closely considered by clinical staff. This systematic review summarized both the premorbid predicting factors and postmorbid outcomes of psychological stress after stroke, which provides coherent references for clinical treatment from the early stage to the late stage.

## Data Availability Statement

The original contributions presented in the study are included in the article/Supplementary Material, further inquiries can be directed to the corresponding author/s.

## Author Contributions

SZ and YY performed data collection, extraction, and assessment, as well as manuscript writing. JL and YW designed the research and search strategy. TX and WZ reviewed the data quality. YX, CT, and JZ contributed to the analysis and explanation of the data. All authors contributed to the manuscript revision and read and approved the submitted version.

## Funding

This study was supported by the National Key Research Program of China (2016YFE0126000), the Key R&D projects of Yangzhou (YZ2020097), the Open project of Key Laboratory of Zoonosis in Jiangsu Province (HX20014), and projects supported by the Six Talent Peaks in Jiangsu Province (WSN-082).

## Conflict of Interest

The authors declare that the research was conducted in the absence of any commercial or financial relationships that could be construed as a potential conflict of interest.

## Publisher's Note

All claims expressed in this article are solely those of the authors and do not necessarily represent those of their affiliated organizations, or those of the publisher, the editors and the reviewers. Any product that may be evaluated in this article, or claim that may be made by its manufacturer, is not guaranteed or endorsed by the publisher.
