# Supplementary material for: Contributing Factors and Induced Outcomes of Psychological Stress Response in Stroke Survivors: A Systematic Review
Source: Front Neurol. 2022 Jun 22;13:843055. doi: 10.3389/fneur.2022.843055 (PMC9257184; doi:10.3389/fneur.2022.843055)
Supplement: Supplementary file 2 [file Table_2.DOCX]

**Supplementary Table 2: Search terms utilized for literature retrieval and Search Strategy**

| **Search terms utilized for literature retrieval** | |
| --- | --- |
| Stroke [MeSH Terms] | Stress, Psychological [MeSH Terms] |
| Strokes | Psychological Stresses |
| Cerebrovascular Accident | Stresses, Psychological |
| Cerebrovascular Accidents | Life Stress |
| CVA (Cerebrovascular Accident) | Life Stresses |
| CVAs (Cerebrovascular Accident | Stress, Life |
| Cerebrovascular Apoplexy | Stresses, Life |
| Apoplexy, Cerebrovascular | Stress, Psychologic |
| Vascular Accident, Brain | Psychologic Stress |
| Brain Vascular Accident | Stressor, Psychological |
| Brain Vascular Accidents | Psychological Stressor |
| Vascular Accidents, Brain | Psychological Stressors |
| Cerebrovascular Stroke | Stressors, Psychological |
| Cerebrovascular Strokes | Psychological Stress |
| Stroke, Cerebrovascular |  |
| Strokes, Cerebrovascular |  |
| Apoplexy |  |
| Cerebral Stroke |  |
| Cerebral Strokes |  |
| Stroke, Cerebral |  |
| Strokes, Cerebral |  |
| Stroke, Acute |  |
| Acute Stroke |  |
| Acute Strokes |  |
| Strokes, Acute |  |
| Cerebrovascular Accident, Acute |  |
| Acute Cerebrovascular Accident |  |
| Acute Cerebrovascular Accidents |  |
| Cerebrovascular Accidents, Acute |  |

| **Search Strategy** | | |
| --- | --- | --- |
| **Database** | **Number of results** | **Search terms** |
| PubMed | 1214 | ((stroke[MeSH Terms]) OR (Strokes[Title/Abstract]) OR (Cerebrovascular Accident[Title/Abstract]) OR (Cerebrovascular Accidents[Title/Abstract]) OR (CVA (Cerebrovascular Accident)[Title/Abstract]) OR (CVAs (Cerebrovascular Accident)[Title/Abstract]) OR (Cerebrovascular Apoplexy[Title/Abstract]) OR (Apoplexy, Cerebrovascular[Title/Abstract]) OR (Vascular Accident, Brain[Title/Abstract]) OR (Brain Vascular Accident[Title/Abstract]) OR (Brain Vascular Accidents[Title/Abstract]) OR (Vascular Accidents, Brain[Title/Abstract]) OR (Cerebrovascular Stroke[Title/Abstract]) OR (Cerebrovascular Strokes[Title/Abstract]) OR (Stroke, Cerebrovascular[Title/Abstract]) OR (Strokes, Cerebrovascular[Title/Abstract]) OR (Apoplexy[Title/Abstract]) OR (Cerebral Stroke[Title/Abstract]) OR (Cerebral Strokes[Title/Abstract]) OR (Stroke, Cerebral[Title/Abstract]) OR (Strokes, Cerebral[Title/Abstract]) OR (Stroke, Acute[Title/Abstract]) OR (Acute Stroke[Title/Abstract]) OR (Acute Strokes[Title/Abstract]) OR (Strokes, Acute[Title/Abstract]) OR (Cerebrovascular Accident, Acute[Title/Abstract]) OR (Acute Cerebrovascular Accident[Title/Abstract]) OR (Acute Cerebrovascular Accidents[Title/Abstract]) OR (Cerebrovascular Accidents, Acute[Title/Abstract])) AND ((Stress, Psychological[MeSH Terms]) OR (Psychological Stresses[Title/Abstract]) OR (Stresses, Psychological[Title/Abstract]) OR (Life Stress[Title/Abstract]) OR (Life Stresses[Title/Abstract]) OR (Stress, Life[Title/Abstract]) OR (Stresses, Life[Title/Abstract]) OR (Stress, Psychologic[Title/Abstract]) OR (Psychologic Stress[Title/Abstract]) OR (Stressor, Psychological[Title/Abstract]) OR (Psychological Stressor[Title/Abstract]) OR (Psychological Stressors[Title/Abstract]) OR (Stressors, Psychological[Title/Abstract]) OR (Psychological Stress[Title/Abstract])) |
| EMBASE | 2313 | ('cerebrovascular accident'/exp OR ('accident, cerebrovascular':ab,ti OR 'acute cerebrovascular lesion':ab,ti OR 'acute focal cerebral vasculopathy':ab,ti OR 'acute stroke':ab,ti OR 'apoplectic stroke':ab,ti OR apoplexia:ab,ti OR apoplexy:ab,ti OR 'blood flow disturbance, brain':ab,ti OR 'brain accident':ab,ti OR 'brain attack':ab,ti OR 'brain blood flow disturbance':ab,ti OR 'brain insult':ab,ti OR 'brain insultus':ab,ti OR 'brain vascular accident':ab,ti OR 'cerebral apoplexia':ab,ti OR 'cerebral insult':ab,ti OR 'cerebral stroke':ab,ti OR 'cerebral vascular accident':ab,ti OR 'cerebral vascular insufficiency':ab,ti OR 'cerebro vascular accident':ab,ti OR 'cerebrovascular arrest':ab,ti OR 'cerebrovascular failure':ab,ti OR 'cerebrovascular injury':ab,ti OR 'cerebrovascular insufficiency':ab,ti OR 'cerebrovascular insult':ab,ti OR 'cerebrum vascular accident':ab,ti OR 'cryptogenic stroke':ab,ti OR cva:ab,ti OR 'ischaemic seizure':ab,ti OR 'ischemic seizure':ab,ti OR stroke:ab,ti OR 'thrombotic stroke':ab,ti)) AND ('mental stress'/exp OR ('mental stresses':ab,ti OR 'mental tension':ab,ti OR 'nervous stress':ab,ti OR 'psychic stress':ab,ti OR 'psychic tension':ab,ti OR 'psycho-social stress':ab,ti OR 'psycho-social stresses':ab,ti OR 'psychologic stress':ab,ti OR 'psychological stress':ab,ti OR 'psychosocial stress':ab,ti OR 'psychosocial stresses':ab,ti OR 'stress, mental':ab,ti OR 'stress, psychologic':ab,ti OR 'stress, psychological':ab,ti OR 'tension, mental':ab,ti OR 'tension, psychic':ab,ti)) |
| Web of science | 1790 | TS=((stroke OR Strokes OR Cerebrovascular Accident OR Cerebrovascular Accidents OR CVA (Cerebrovascular Accident) OR CVAs (Cerebrovascular Accidents) OR Cerebrovascular Apoplexy OR Apoplexy, Cerebrovascular OR Vascular Accident, Brain OR Brain Vascular Accident OR Brain Vascular Accidents OR Vascular Accidents, Brain OR Cerebrovascular Stroke OR Cerebrovascular Strokes OR Stroke, Cerebrovascular OR Strokes, Cerebrovascular OR Apoplexy OR Cerebral Stroke OR Cerebral Strokes OR Stroke, Cerebral OR Strokes, Cerebral OR Stroke, Acute OR Acute Stroke OR Acute Strokes OR Strokes, Acute OR Cerebrovascular Accident, Acute OR Acute Cerebrovascular Accident OR Acute Cerebrovascular Accidents OR Cerebrovascular Accidents, Acute) AND (Stress, Psychological OR Psychological Stresses OR Stresses, Psychological OR Life Stress OR Life Stresses OR Stress, Life OR Stresses, Life OR Stress, Psychologic OR Psychologic Stress OR Stressor, Psychological OR Psychological Stressor OR Psychological Stressors OR Stressors, Psychological OR Psychological Stress)) |
| CNKI | 230 | TKA=(脑卒中 + 中风 + 脑血管意外 + 急性脑卒中 + 急性中风 + 急性脑血管意外) AND TKA=(心理应激 + 心理压力 + 生活应激 + 生活压力) |
| WanFangData | 639 | 题名或关键词:(脑卒中 OR 中风 OR 脑血管意外 OR 急性脑卒中 OR 急性中风 OR 急性脑血管意外) AND 题名或关键词:(心理应激 OR 心理压力 OR 生活应激 OR 生活压力) |
| CQVIP | 64 | M=(脑卒中 OR 中风 OR 脑血管意外 OR 急性脑卒中 OR 急性中风 OR 急性脑血管意外) AND M= (心理应激 OR 心理压力 OR 生活应激 OR 生活压力) |
